# Supplementary material for: Protein structure determination in human cells by in-cell NMR and a reporter system to optimize protein delivery or transexpression
Source: Commun Biol. 2022 Dec 2;5:1322. doi: 10.1038/s42003-022-04251-6 (PMC9718737; doi:10.1038/s42003-022-04251-6)
Supplement: Supplementary file 2 — Supplementary Materials [file 42003_2022_4251_MOESM2_ESM.docx]

­Protein structure determination in human cells by in-cell NMR and a reporter system to optimize protein delivery or transexpression

Juan A. Gerez^1*^, Natalia C. Prymaczok^1^, Harindranath Kadavath^1^, Dhiman Ghosh^1^, Matthias Bütikofer^1^, Yanick Fleischmann^1^, Peter Güntert^1,2,3^, Roland Riek^1*^

* to whom correspondence should be addressed to juan.gerez@phys.chem.ethz.ch Tel: +41 44 633 66 27, roland.riek@phys.chem.ethz.ch Tel: +41 44 632 61 39

**This PDF file includes:**

Supplementary Figures 1 to 4

Supplementary Table 1

**
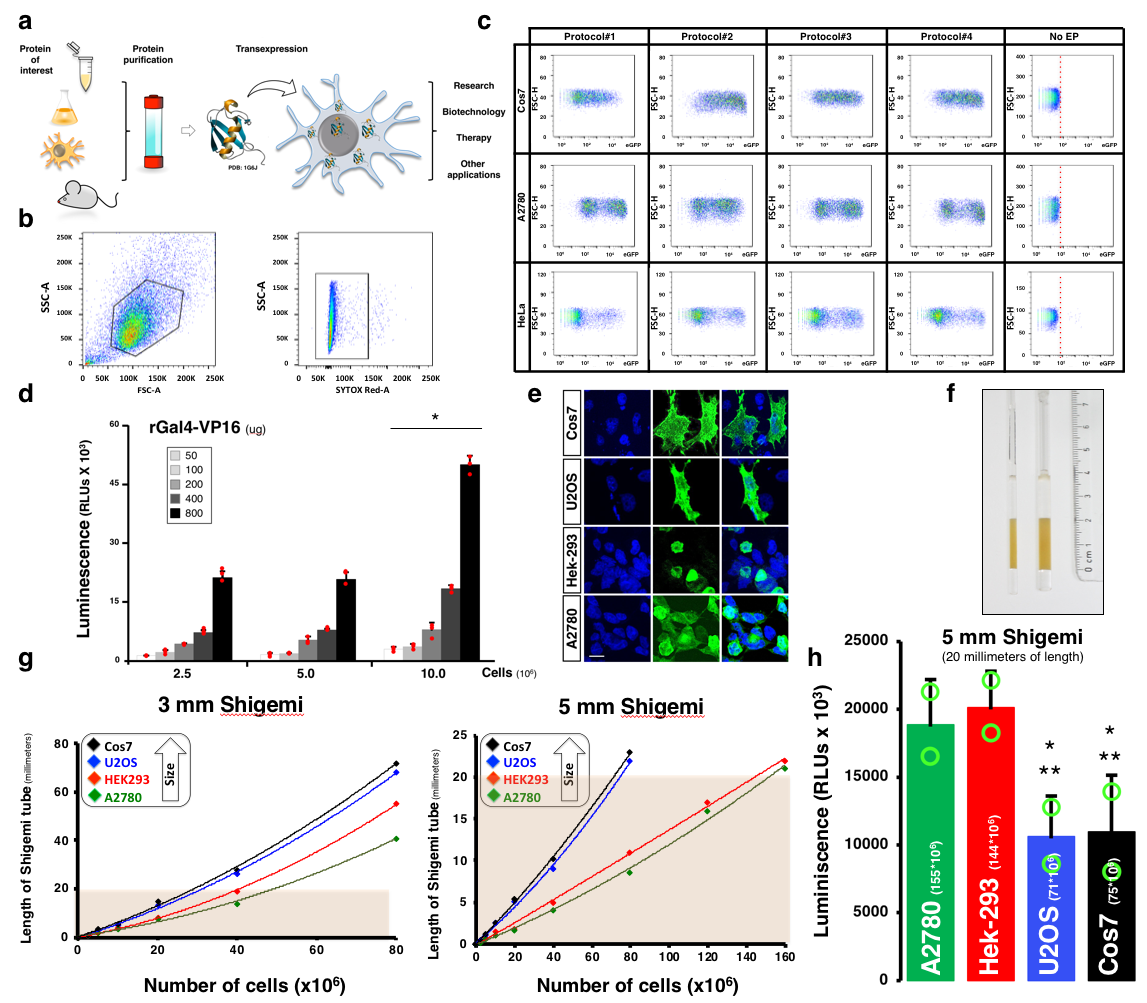
**

**Supplementary Figure 1. Transexpression and its reporter system. a**) Representative cartoon of transexpression. Transexpression is defined here as the delivery of an exogenous protein into mammalian cells. The protein necessarily needs to be obtained from an external source such as *in vitro* translation or heterologous expression systems that include bacteria, mammalian cells as well as isolated tissue and animals (left). The purified protein (middle) is then “transexpressed” into mammalian cells by any of the experimental methods for protein delivery available. The protein is thus incorporated to the proteome of the recipient cells which can be used for research or any other application (right). **b** and **c**) Four different protocols differing on the buffer composition were used to transexpress by electroporation rGal4-VP16 into Cos7, A2780 and HeLa cells. Fluorescence was quantified by fluorescence-activated cell sorting and the cell populations selected for eGFP signal quantification are shown on the right. **d**) Protein amount and cell number dependence on transexpression by electroporation. Increasing amounts of cells (2.5 to 10 million) were used to transexpress increasing amounts of rGal4-VP16 (from 50 to 800 µg). Bioluminescence was then quantified in these cells as they contained a related reporter gene called pG5-Luc encoding the firefly luciferase. The bar plot shows the luminiscence values of 10 million cells (*n* = 3 biologically independent samples) electroporated with the different amounts of rGal4-VP16. The results are expressed as means + SD. **p* < 0.05 [one-way analysis of variance (ANOVA), followed by Dunnett’s post hoc test]. **e**) Fluorescence microscopy of eGFP-expressing Cos7, U2OS, Hek-293 and A2780 cells. These cells display different sizes (scale bar 10 µm). **f**) Mammalian cells are collected in NMR tubes of 3 and 5 millimeters of diameter and packed by applying a soft centrifugation of 300 x g for 2 minutes. Only around 2 centimeters of the packed cells are used for NMR measurements. **g**) Different amounts of Cos7, U2OS, Hek-293 and A2708 cells were collected in 3 millimeters (left) of 5 millimeters (right) NMR tubes and then packed by a centrifugation step. The graph shows the volume occupancy (measured as the length of the tube occupied by the obtained cell pellet) as a function of cell number. The brown boxes are the active volumes for NMR measurements (around 2 centimeters of longitudinal axis of the tubes). **h**) A fixed volume of cell pellet, corresponding to 2 centimeters of the NMR tube, was obtained with A2780, Hek-293, U2OS and Cos7 cells transexpressed with rGal4-VP16. Bioluminescence was then quantified in these cells as they contained the pG5-Luc plasmid. The bar plot shows the luminescence values of 10 million cells (*n* = 2 biologically independent samples) electroporated with the different amounts of rGAL4-VP16. The results are expressed as means + SD. **p* < 0.05 [one-way analysis of variance (ANOVA), followed by Dunnett’s post hoc test].


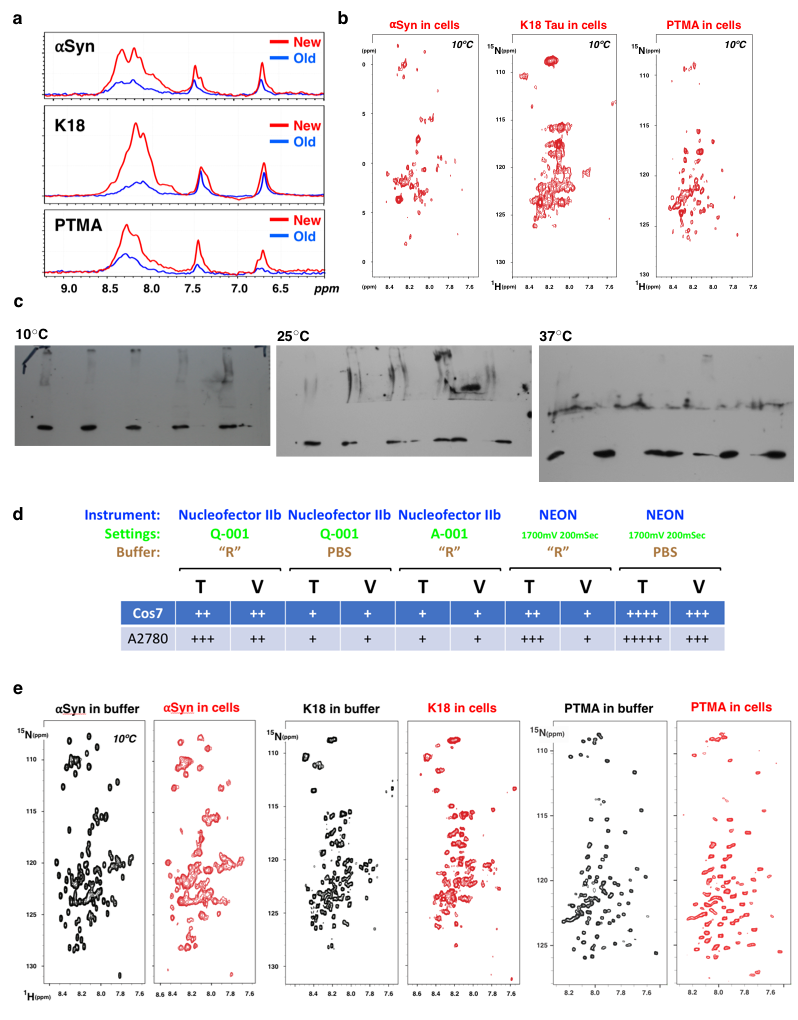

Supplementary Figure 2. In-cell NMR of IDPs. a) 1D HMQC NMR experiments of ^15^N-labeled alpha-synuclein (⍺Syn), K18 tau and prothymosin-alpha (PTMA) transexpressed in mammalian cells using the new protocol (in red) and the previously published in-cell NMR method (in blue). Because good quality spectra are obtained only with the new method (see Fig. 2d and Supplementary Fig. 2b), and because compared to the old method the new protocol requires reduced amounts of ^15^N-labeled protein, we concluded that the new method yields a more efficient transexpression. b) 2D HMQC NMR experiments of ^15^N-labeled ⍺Syn, K18 and PTMA transexpressed using the old method with the same amount of recombinant proteins used in Fig. 2d and S2e. c) Uncropped gels of the image of Fig. 2c. d) Summary of five representative transexpression protocols used in this work. Two cell lines (Cos7 and A2780) were tested using electroporator devices of two manufacturers; Nucleofector-IIb (AMAXA-LONZA) and Neon (Invitrogen). The settings for the AMAXA electroporator were fixed to protocols Q-001 and A-001, while for the Invitrogen electroporator were 1700 mV and 200 mSec. The buffers used were buffer “R” (AMAXA-LONZA) and PBS. Transexpression (denoted with a “T”) and cell viability (denoted with a “V”) were assayed with the rGAL4-VP16 reporter system and the trypan blue test, respectively. e) 2D HMQC NMR experiments of ^15^N-labeled ⍺Syn, K18 and PTMA transexpressed in mammalian cells using the new in-cell NMR protocol (red). The references were acquired with the same proteins in buffer (black). f) Residue-resolved backbone amide NMR signal attenuation of αSyn and K18 tau in mammalian cells. Some structural features are of these proteins shown in the bottom. *I*_rel_ = *I_cells_*/*I*_buffer_ , relative intensity (*I*_rel_) of the high of the NMR cross-peak in cells (*I_cells_*) divided by the high of the NMR cross-peak in buffer (*I*_buffer_). g) 2D HMQC NMR experiments of ^15^N-labeled ⍺Syn transexpressed in mammalian cells using the new in-cell NMR protocol and kept in the NMR tube for 4, 8, 12 and 16 hours. h) Spectra overlap of selected areas of the spectra of panel g).

**
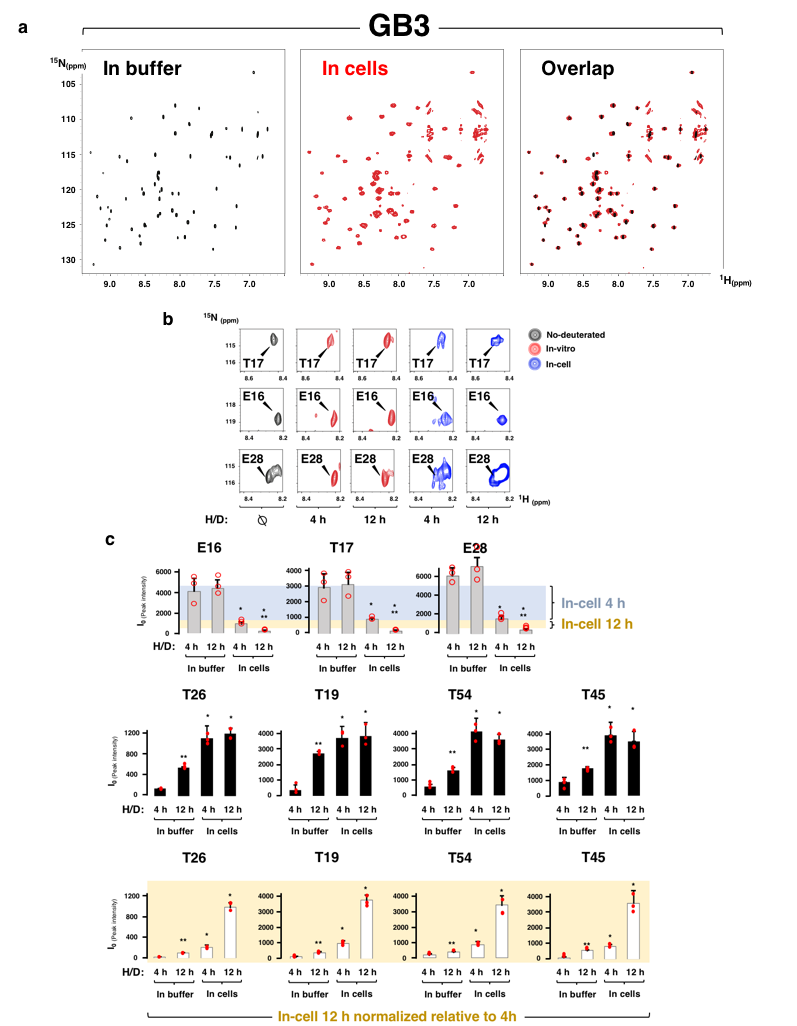
**

**Supplementary Figure 3. In-cell NMR of folded proteins. a** and **b)** Hydrogen-deuterium experiments (H/D) on GB1 in buffer and in cells. **a**) Selected resonances (T17, E16 and E28) of the two dimensional [^15^N,^1^H]-HMQC NMR experiments of non-deuterated (black) and deuterated (red and blue) ^15^N-labeled GB1. H/D exchange was allowed to occur in buffer (red) or in cells (blue), and at two time points, 4 h and 12 h. In buffer, the intensity of these three residues is similar to non-deuterated GB1 and do not change with time indicating that a full H/D exchange has occurred (see also panel b). **c**) Bar plot showing the residue-resolved backbone amide NMR signal intensity of deuterated GB1 in buffer and in mammalian cells at the two time points. Upper part: the resonances of E16, T17 and E28 were used to estimate the cell effect at 4 and 12 h. At 4 h resonances intensities are lower for the in-cell samples due to lower concentration of transexpressed GB1 in cells. This reduction of peak intensities is indicated by a blue box. In cells at 12 h the peak intensity is lower than at 4 h. This effect is due to processing and turnover of this protein in cells. This effect is indicated by an orange box. Middle part: resonances of T26, T19, T45 and T54. Lower part: the peak intensities of T26, T19, T45 and T54 in cells at 12 h were normalized relative to 4 h. To calculate the normalization factor the peak intensities in cells at 4 h of E16, T17 and E28 were used. * and **, *p* < 0.05 compared to in buffer and 4 h, respectively. (*n* = 3 biologically no independent samples).


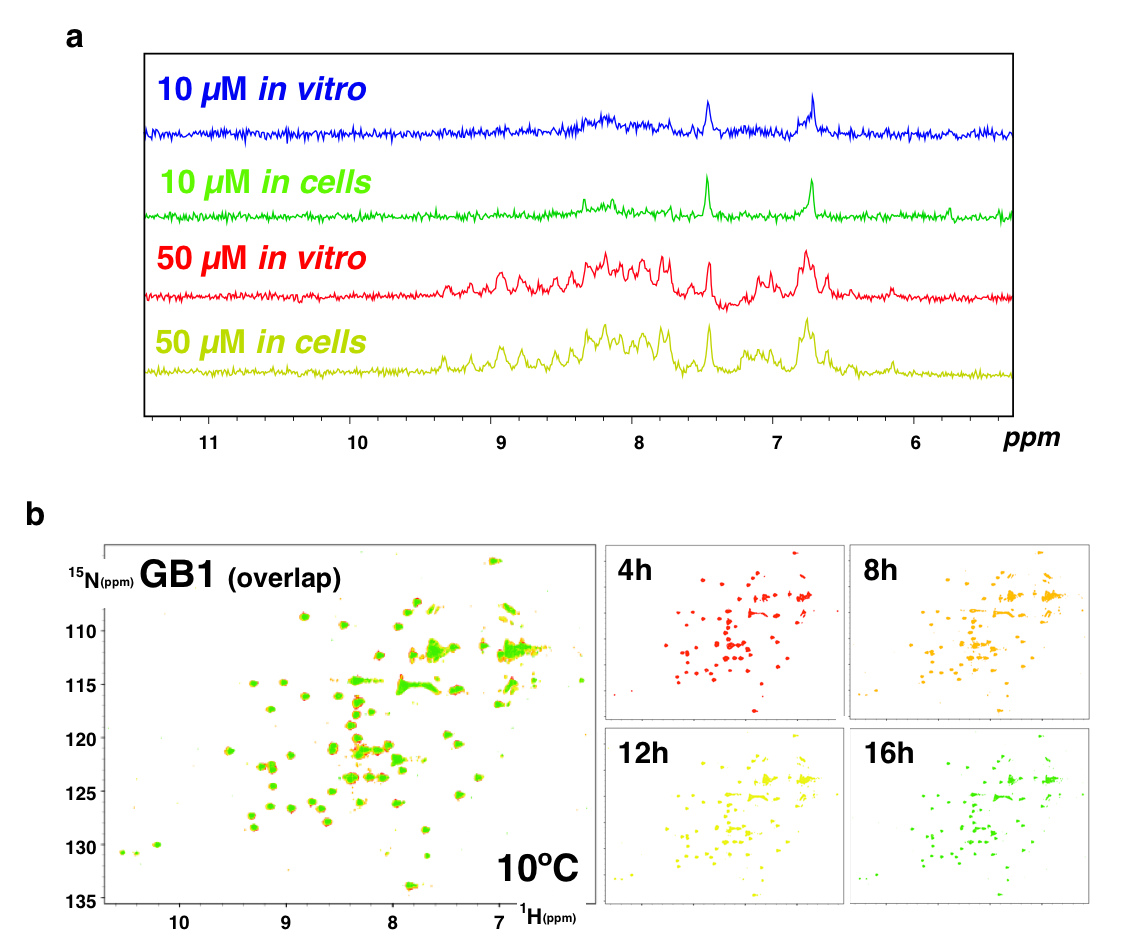


**Supplementary Figure 4. Structure determination in mammalian cells. a)** Quantification of GB1 protein levels by NMR. 1D HMQC NMR experiments of ^15^N-labeled GB1 *in vitro* (blue and red) and transexpressed in mammalian cells (green and yellow). The concentration of the samples *in vitro* is known and were used to determine the concentration of GB1 in cells. **b**) HMQC NMR experiments of ^15^N-labeled GB1 transexpressed in mammalian cells and kept in the NMR tube at 10 ^○^C for 4, 8, 12 and 16 hours. Spectra overlap is shown on the left, while each individual spectrum is shown on the right.
