# Supplementary material for: Protein structure determination in human cells by in-cell NMR and a reporter system to optimize protein delivery or transexpression
Source: Commun Biol. 2022 Dec 2;5:1322. doi: 10.1038/s42003-022-04251-6 (PMC9718737; doi:10.1038/s42003-022-04251-6)
Supplement: Supplementary file 3 — Description of Additional Supplementary Files [file 42003_2022_4251_MOESM3_ESM.pdf]

Description of Additional Supplementary Files

File name: Supplementary Data 1

Description: Source data for the plots of the manuscript "Protein structure determination in human cells by in-cell NMR and optimized protein delivery or transexpression" by Gerez et. al.
